# Editorial: Liquid metal-based flexible bioelectronics and biointerfaces

**DOI:** 10.3389/fbioe.2023.1254744

**Published:** 2023-08-02

**Authors:** Liangtao Li, Liang Hu

**Affiliations:** Beijing Advanced Innovation Center for Biomedical Engineering, School of Biological Science and Medical Engineering, Beihang University, Beijing, China

**Keywords:** liquid metal, flexible electronics, fabrication technique, wearable device, surface modification

Gallium-based liquid metals have presented fascinating properties including high electrical conductivity, excellent fluidity at room temperature, favorable biocompatibility and intriguing interfacial characteristics, which are especially suitable for flexible electronics and interfaces. Recently liquid metal-based electronics and devices have been used for biomedical applications such as bioelectronic systems and biomedical sensing for physiological signal monitoring. Underlying these applications, liquid metal-related researches from diverse perspectives have been carried out from the physio-chemical properties, patterning, molding and assembly techniques to device design and integration, which are also revealed in the present Research Topic. In anticipation of the future developments in this field, we compiled this Research Topic to provide latest advancements and perspectives on *Liquid metal-based flexible bioelectronics and biointerfaces*. This Research Topic includes two reviews and four original research articles, which range from basic material physics, manufacture techniques to bioelectronic applications.

As the modulation of the surface tension is the key in manipulating liquid metals for further fabrication process, Wang et al. report the effect of ratchet graphite substrate on the electrically driven heartbeat effect of LM droplet. The beating frequencies can be tuned by the asymmetrical sawtooth structures of the graphite electrode, which is basically due to the reversible electrochemical oxidation of the LM surface in the NaOH solution. This study can offer inspiration to a wide range of bioelectronics applications including intellectual switches, micro actuators and etc.

The original works from Yan and Li focused on the liquid metal microfluidic-related fabrication techniques. Yan et al. reported a facial method to obtain free-standing gallium mold and to form 3D flexible microfluidic channels with the combination of elastomers such as PDMS and Ecoflex. The study presents a conceptual demonstration of wearable microfluidic cooler with liquid metal used as a coolant. Moreover, this 3D microfluid can be easily used as flexible and wearable electronic devices such as stress sensors, flexible circuits, etc. as reported by many other studies (Guo et al., 2019; Ren et al., 2019; Yao et al., 2019; Wang et al., 2020). Li et al. reported an enhanced alignment bonding technique for microfluidics especially helpful for multi-layer microfluidics. These works can shed light on the liquid metal microfluidic-based applications such as electromagnetic pumps for drug delivery as reported also by their group.

It is known that the softer and thinner the flexible electronics are, the better conformability and signal quality they can achieve. The works from Li and two reviews from Guo’s and Tang’ Group intensively discussed the liquid metal-based soft bioelectronics. Ping et al. reviewed the latest advances in liquid metal-enabled conformal electronics, especially on the printing methods as well as the bioelectronic devices. The printing methods for liquid metal or its composite materials are usually convenient and low-cost, similar to most on-desk printing techniques, which means it is easy to generalize. It also summarized the representative liquid metal-enabled conformal electronic devices including self-healing devices, degradable devices, flexible hybrid electronic devices and variable stiffness devices and multi-layer large area circuits. The challenges can lie in the relatively high cost of gallium and the conformal and precise interconnection between rigid electronic components with LM wires. However, combined the low stiffness close to ∼0 Young’s Modulus with excellent electrical conductivity superior to most soft conductors, LM still presents great promise in flexible and soft electronic that can fully conformal to skin and even to tissue.

Apart from the softness, the air permeability is another consideration for wearable devices as long-time physiological monitoring. From this perspective, Yang et al. reviewed the recent advancement of fiber-based soft electronics enabled by liquid metals, which combines liquid metal and spinning technology together. Two main strategies to fabricate breathable liquid metal electronics include: 1) patterning liquid metal on fiber membranes, 2) preparing liquid metal fibers and weaving them into breathable e-textiles. Usually, these fiber-based electronics are soft and breathable, but challenges such as non-sticky to skin and difficulty in encapsulation still exist.

To overcome the challenges mentioned, the work reported by Li et al. adopted the second strategy and develop a stick and porous LM@ PU/CNT/LM membrane for soft and stretchable wearable electronics. Fabricated by electrospinning and CNT coating, this film is antibacterial, air permeable, adhesive to skin and electrically conductive, which is excellent for wearable electronics. Thus it is used as a strain sensor with high sensitivity (GF 3 at 50% strain and 14 at 400% strain) to monitor motion signals mainly due to the stretchability of PU. It can be also used to monitor ECG/EMG signals as soft electrodes, which is well conformable to skin.

In addition, a series of research work has been carried out on gallium-based liquid metals in chemical sensing. First of all, gallium tends to form a thin oxide layer on its surface, which can be temporarily removed by acidic or alkaline solutions. Based on this phenomenon, the oxide film itself can be used as a sensing element. Wang et al. (2022) presented a study to measure the pH of acidic or alkaline solutions by morphological changes caused by the increase in the surface tension of the liquid metal during the oxide film’s removal. However, in most cases, the oxide film creates a barrier to biological applications by interfering with electrochemical measurements and altering the physicochemical properties of the surface, thereby affecting its electrical properties. Acidic or alkaline solutions do not prevent further oxidation of the gallium surface. Therefore, the liquid metal surface modification to achieve a stable interface for biological applications is essential. Lim et al. (2022) encapsulated PEDOT:BF4 nanostructures on the interface of a liquid metal flexible electrode by electrochemical deposition, which not only prevented the oxidation and degradation of the liquid metal, but also significantly improved the electrochemical performance of the liquid metal electrode. With this electrode, a gallium-based liquid metal bioelectronic device was used for the first time to perform single-unit neural recordings in awake behaving non-human primates and showed stable and high-quality recording results. In addition to liquid metal bulk, nanoparticles have more flexible construction schemes for practical bioelectronic applications, but surface modification of them is a greater challenge. Huang et al. (2022) modified the surface of EGaIn with p-aniline derivatives to form stable spherical nanoparticles, which endowed the nanoparticles with strong electrical conductivity, stability and oxidation resistance. Among them, the EGaIn-PABA-AuNPs-InsApt sensing interface formed by modifying structure-switching aptamer targeting insulin on EGaIn-PABA (p-aminobenzoic acid) realized the electrochemical detection of insulin with the limit of detection (LOD) of 0.38 pM, which demonstrated the great potential of gallium-based liquid metals in the field of medical treatment.

In conclusion, from the research works and reviews included in this Research Topic, it is revealed that liquid metal-based electronics and devices can be either present in the form of fluid within microfluidics, or as conductive ink via molding on/in various 2D or 3D matrix materials. Fabrication technologies have evolved rapidly to create liquid metal structures on 2D and 3D surfaces or within matrixes. These techniques form the basis for the fabrication of functional electronic devices. To further promote the development of these devices in bioelectronics, more liquid metal-compatible electronic materials and assembly techniques are in great demand. In addition, at this stage, the applications of liquid metals in flexible bioelectronics are more conceptual than practical. Further studies are expected to develop those liquid metal-based flexible devices with better stability and functionality in practical scenarios.
